# Exploring perinatal care and birth experiences in women with visual impairment: A retrospective study

**DOI:** 10.3892/mi.2024.190

**Published:** 2024-08-23

**Authors:** Chrisanthi Makeroufa, Athina Diamanti

**Affiliations:** Department of Midwifery, Faculty of Health and Care Sciences, University of West Attica, 12243 Athens, Greece

**Keywords:** visual impairment, perinatal care, accessible healthcare, pregnancy, healthcare professionals

## Abstract

Globally, ~2.2 billion individuals suffer from visual impairment, with a large proportion of these individuals being women of reproductive age. This demographic often faces unique healthcare challenges, particularly during pregnancy, childbirth and the puerperium. However, despite the significant prevalence of visual impairment among women, there are only a limited number of studies available addressing their specific perinatal care needs. The present study aimed to fill this gap by exploring the perinatal experiences of women who are visually impaired, highlighting the existing care provisions and identifying areas for improvement. For this purpose, a retrospective study was conducted from January to June, 2021, involving 22 women with visual impairment who gave birth after 2005. The study participants were recruited through several organizations supporting individuals who are visually impaired and the participants completed a comprehensive electronic questionnaire designed to be accessible for individuals with visual impairments. The questionnaire covered demographical data, pregnancy, childbirth, puerperium period experiences and interactions with healthcare professionals. The participants included in the present study ranged in age from 29 to >35 years. The origins of their total or partial blindness varied. As shown by the results, ~45.5% of the participants considered they received equivalent levels of midwifery and gynecological care compared to women without visual impairments, and half of the participants reported that midwives and gynecologists were willing to provide such care. However, the majority (90.9%) indicated a lack of adequate knowledge among healthcare providers regarding the specific perinatal care needs of women who are visually impaired. These findings underscore the critical need for the specialized training for healthcare providers and the development of more inclusive, accessible healthcare practices to improve perinatal care for women who are visually impaired.

## Introduction

A report by the World Health Organization (WHO), as well as previous studies by the have revealed that at least 2.2 billion individuals globally, primarily due to population growth, suffer from vision impairment or blindness, with a great proportion of these individuals being women. Notably, a number of these impairments are age-related, necessitating focused attention on women of reproductive age (1-3). In Europe alone, ~2.4% of women within this demographic are significantly visually impaired, underscoring an urgent need for specialized healthcare services tailored to their unique needs (4).

Historically, societal perceptions have marginalized the sexual and reproductive desires of individuals with disabilities, often labeling these aspirations as non-existent or perilous. Yet, the inherent right to parenthood persists universally, advocating that all individuals, irrespective of disabilities, deserve to experience this fundamental aspect of human development (3,5). Despite considerable research being made into the general lack of awareness among healthcare professionals (HCPs) about the specific needs of this population group, studies focusing directly on women who are visually impaired remain limited. This knowledge gap perpetuates social stigma and results in suboptimal health counseling for these women, impacting their overall quality of life (5-9).

Despite the introduction of legal protections for individuals with disabilities in the 20th century, practical barriers still obstruct their access to healthcare services (10,11). Transportation to medical facilities and inappropriate behavior from healthcare providers are common daily challenges. Studies have indicated that women with visual impairments are more likely to face complications during pregnancy and have adverse birth outcomes (5,12-15). However, customizing childbirth preparation classes to meet their individual needs can significantly mitigate these risks. Providing psychosocial support and personalized sessions are essential steps toward fulfilling this goal (6,16).

It is evident that the existing perinatal care systems are ill-equipped to adequately support women who are visually impaired (6,8,14). Public health objectives emphasize improving health outcomes across all populations, highlighting the necessity of accessible information and inclusive services to diminish health disparities and enhance life quality for these women (8,17). Despite their legal right to motherhood, visually impaired women often face undue prejudice, viewed as incapable of experiencing normal childbirth or fulfilling maternal roles effectively (3,16).

The Royal College of Midwives emphasizes the importance of supporting mothers with disabilities by adhering to established guidelines [https://www.rcm.org.uk/media/4521/rcm_position-statement_multiple-disadvantaged_draft_final.pdf]. Midwives must not only be aware of their responsibilities, but must also be equipped to provide adaptable, innovative and empathetic care, tailored to the unique challenges faced by these women, thereby ensuring positive experiences throughout pregnancy, childbirth and motherhood (18).

In recent years, numerous studies have highlighted the critical need for maternal care that inclusively supports all women, particularly those from vulnerable groups (16-18). As such, it is paramount that Greece intensifies its efforts to scrutinize and enhance the level of care provided to its marginalized populations through detailed assessments such as questionnaires, interviews and case studies. Currently there are a handful of studies exploring perinatal care and birth experiences in women with visual impairment (6,16). Notably, while a number of these impairments are due to conditions, such as unmet refractive error or cataract, which are generally reversible, the present study focuses on the subset of visual impairments that are not easily corrected and persist into or arise during reproductive age. These include conditions, such as glaucoma, retinitis pigmentosa and congenital vision disorders. To the best of our knowledge, the present study is the first study from Greece to investigate these aspects in women with visual impairment. The present study aimed to highlight the importance of developing guidelines that improve healthcare quality, the of proficiency healthcare providers and the availability of adequate facilities, as derived from the antenatal population with any degree of visual impairment experiences during pregnancy, childbirth and puerperium.

## Patients and methods

### Study design and population

The present descriptive retrospective study was conducted between January and June, 2021, focusing on women in Greece with visual impairments who gave birth after 2005. The cohort consisted of 22 women who were either totally or partially blind, mostly from Athens. Participants ranged in age from 29 to 44 years, with a median age of 32 years.

### Inclusion criteria

The present study used the following inclusion criteria for the recruitment of patients: i) Age: Women had to have an age ≥18; ii) pregnancy period: Women who were pregnant between the years 2005 and 2021 were included; iii) visual impairment: Women with documented visual impairment, as defined by best corrected visual acuity (BCVA), electroretinogram (ERG) or impaired visual fields were included; iv) medical documentation: Participants had to provide medical documentation confirming their visual impairment diagnosis from a certified ophthalmologist; v) support from Greek organizations: Women who had been supported by a Greek organization for individuals with visual impairment (e.g., National Federation for the Blind) for a minimum of 6 months were included; vi) residency: Women had to be residing in Greece at the time of their pregnancy; and vii) consent: Women had to be willing and able to provide informed consent to participate in the study.

### Exclusion criteria

The exclusion criteria used herein were the following: i) A lack of medical documentation: Women who could not provide medical documentation confirming their visual impairment were not included; ii) women with inadequate levels of visual impairment were excluded; iii) insufficient duration of support: Women who had not been supported by a Greek organization for individuals with visual impairment for at least 6 months were excluded; iv) non-residency: Women who do not reside in Greece or who were not residing in Greece during their pregnancy.vi) inability to provide consent: Women who were unable or unwilling to provide informed consent to participate in the study were not included.

### Recruitment of participants

The study participants were recruited from several organizations dedicated to supporting individuals with visual impairments, including the National Federation for the Blind in Athens (https://www.eoty.gr/the-main-goals-and-activities-of-the-national-federation-of-the-blind-e-o-t), the Panhellenic Association of the Blind-Regional Association of the Blind in Western Greece (https://www.pst.gr), the Training & Rehabilitation Center for the Blind in Thessaloniki (https://keat.gr/?lang=en), ‘Magnites Tifloi’ in Volos (https://www.maty.gr), the Panhellenic Association of Parents & Guardians of People with Serious Vision Problems ‘HERA’ (https://amimoni.gr/en/amimoni-2), and the Pancretan Association of Parents & Friends of Blind and Visually Impaired Children (https://vivliopoleiopataki.gr/persons/view/detail/persons/54779-pagkritios-sillogos-goneon-ke-filon-pedion-tiflon-i-me-miomeni-orasi). These organizations played a crucial role in facilitating access to this uniquely situated group of women, ensuring a representative sample of the visually impaired population undergoing maternity experiences in Greece.

### Research questionnaire

The research tool was a comprehensive questionnaire consisting of 53 questions divided into five distinct sections, as outlined in Data S1 (16-19). The sections included following: i) Demographical and clinical characteristics: This section contained 10 questions aimed at gathering basic and health-related information; ii) pregnancy period: Comprising 22 questions, this section delved into the details of the pregnancy experiences of women; iii) childbirth: This segment included eight questions focused on the labor and delivery experiences; iv) puerperium period: This section also included eight questions, addressing the postpartum experiences; v) health professional and care evaluation: The final five questions assessed the perceptions and interactions of the participants with healthcare providers, as well as the quality of obstetric and gynecological care received.

Participants typically completed the questionnaire within a period of 20 to 25 min. The collection of data was based on self-administered electronic questionnaires filled out by the participants, reflecting their direct experiences. In order to ensure the comprehensive coverage of the topic, the questionnaire design drew on previous studies and relevant scientific literature (14-18).

The survey was administered electronically using Google Forms, which is compatible with various accessibility tools commonly used by visually impaired individuals. To ensure that the questionnaire was fully accessible, it was designed to support screen readers and text-to-speech software, allowing for the auditory reading of the questions. Additionally, the questionnaire was optimized for high contrast and large text settings to cater to individuals with partial vision.

Participants were informed beforehand about the available accessibility features and instructions on how to utilize them effectively were provided. This approach ensured that all participants, regardless of their level of visual impairment, could navigate the questionnaire independently and securely.

The survey interface was tested with multiple types of visual impairment adaptive technologies prior to deployment, acknowledging the diverse needs of the group of participants. This pre-testing phase involved participants from the target demographic who utilized screen readers, braille output devices and magnification software to provide feedback on the accessibility of the survey. Adjustments were made based on their inputs to ensure that the survey was comprehensible and user-friendly for individuals with varying degrees of visual impairment.

In order to ensure the accuracy and reliability of the self-administered data collection process, several measures were implemented, as follows: i) Initial instructions: Participants received detailed instructions on how to complete the survey using Google Forms, which included information on using screen readers, text-to-speech software, and magnification tools. ii) Accessibility features: The Google Forms questionnaire was designed to be fully accessible to individuals with visual impairments. This included compatibility with screen readers, high contrast settings, large text options and voice input capabilities. iii) Family caregiver involvement: While the primary goal was for participants to complete the survey independently, it was acknowledged that some participants may require assistance. In such cases, family caregivers were permitted to assist the participants. However, this assistance was strictly limited to navigating the technology and reading the questions aloud. Caregivers were instructed not to influence or input responses to ensure that the data accurately reflected the experiences and opinions of the participants. iv) Verification of self-administration: In order to further ensure the integrity of the data, a section was included in the survey where participants indicated whether they completed the survey independently or with assistance. For those who received assistance, details on the nature of the assistance provided were requested. v) Pilot testing: Prior to full deployment, the survey underwent pilot testing with a subset of visually impaired individuals. This testing aimed to identify any potential challenges in self-administration and allowed for any necessary adjustments to be made based on feedback. Participants in the pilot test confirmed that the accessibility features were adequate for independent completion. vi) Ethical oversight: The present study was closely monitored by the Ethics Committee of the University of West Attica (Athens, Greece), ensuring adherence to the highest ethical standards. Informed consent was obtained from all participants, emphasizing the importance of honest and independent responses.

Throughout the survey design and implementation, ethical considerations were paramount throughout the design and implementation of the survey. Informed consent was obtained from all participants after providing a detailed explanation of the purpose and methodology of the study, which included measures to maintain privacy and data security. The present study received approval from the Committee of the Midwifery Department of the University of West Attica and was closely monitored during its execution by a three-member committee. The protocol number of the Research Ethics Boards of the University of West Attica was 20/27-09-2019, ensuring adherence to the highest standards of research ethics and participant confidentiality.

### Data analysis and presentation

As the present study was a descriptive study and not an analytical study, no inferential statistics were used. Categorical variables are presented as absolute numbers (frequency in percentages).

## Results

### Demographic and clinical characteristics

All the demographic and clinical characteristics of the study participants are presented in Table I. The present study enrolled 22 women residing in Greece, aged between 29 and >35 years, who consented to participate in the research. Among these patients, 86.4% (n=19) were >35 years of age, 9.1% (n=2) were aged 30 to 34 years, and 4.5% (n=1) were between 25 and 29 years of age. The marital status of the participants varied: 68.2% (n=15) were married, 13.6% (n=3) were single, 9.1% (n=2) were divorced, 4.5% (n=1) were widowed, and another 4.5% (n=1) were estranged. Half of the participants (50%, n=11) had one child. The educational level of the participants was high, with 54.5% (n=12) having received higher education, and 40.9% (n=9) were employed, either in the public or private sector or were self-employed. As regards the annual family income distribution of the participants, 68.2% (n=15) earned between €7,000 and €20,000, and 31.8% (n=7) earned above €20,000. Of the 22 participants, 16 (72.7%) resided in urban areas, specifically in Athens, while 6 (27.3%) were from rural areas across Greece. Among the participants, 15 women (68.2%) received their perinatal care through public healthcare services, while 7 women (31.8%) utilized private healthcare services (Table I).

**Figure 1:**
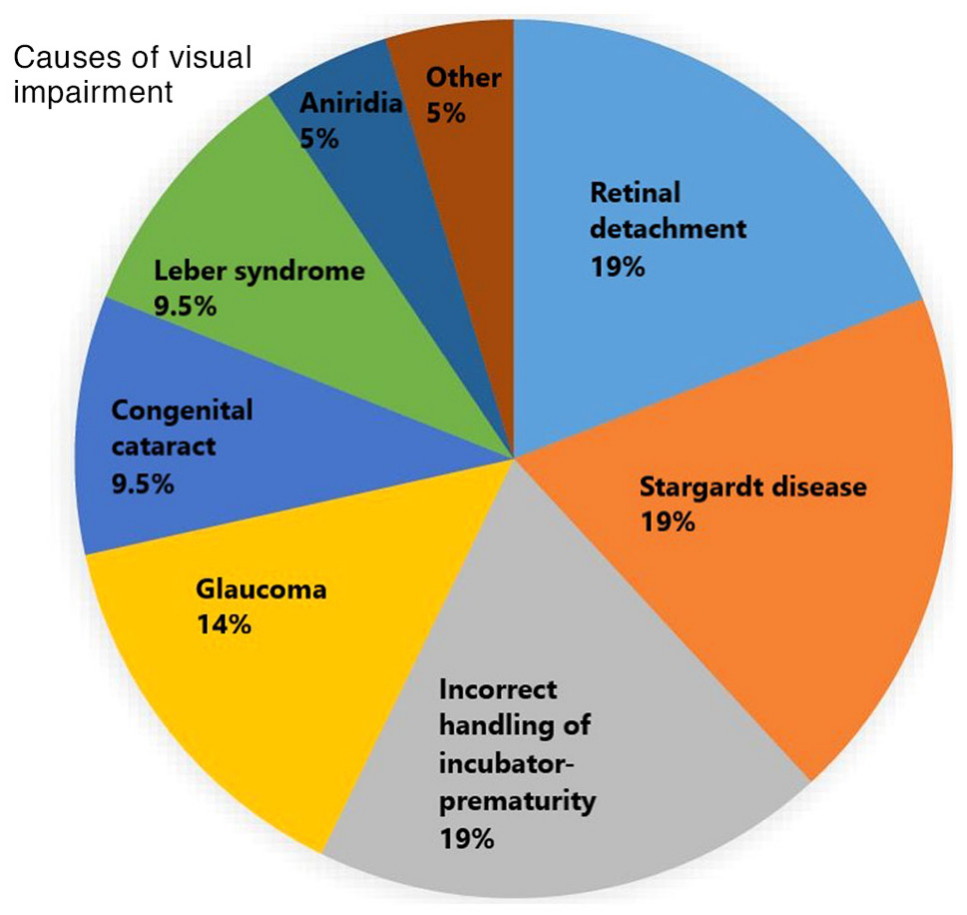
Causes of visual impairment.

All participants had vision impairments, either total or partial blindness. Of these, 63.6% (n=14) were totally blind, and 36.4% (n=8) were partially blind. As regards the onset of blindness, 45.5% (n=10) were congenitally blind, while 54.5% (n=12) acquired blindness later in life due to various causes detailed in Fig. 1. Additionally, 59.1% (n=13) reported that their husbands had a disability, including blindness (Table I).

### Pregnancy and clinical outcomes

The present study cohort consisted of women who had conceived after 2005, with all initial examinations occurring during the first trimester. Among these women, 72.7% (n=16) had planned their pregnancies, and 9.1% (n=2) had utilized assisted reproduction technologies, such as *in vitro* fertilization (IVF). Not all pregnancies resulted in childbirth, with one instance (4.5%) of miscarriage or abortion reported (Table I).

Prior to pregnancy, 6 (27.3%) of the participants were smokers, with a reduction during pregnancy where 3 participants continued smoking at reduced rates. Alcohol consumption was minimal, with 63.6% of participants reporting occasional use prior to pregnancy and only 1 participant (4.5%) consuming a glass every 3 months during pregnancy. None of the participants reported using drugs (Table I).

The participants experienced various changes in weight during pregnancy, with 90.9% (n=20) reporting a weight gain of 5 to 12 kg. However, there were also instances of weight loss and substantial weight gain.

The survey also highlighted the familial and medical support received during pregnancy. The majority, 68.2% (n=15) of the participants, felt well-supported by their husbands, and 81.8% (n=18) reported positive interactions with midwives who supported their pregnancy announcements. Concerning prenatal care, 72.7% (n=16) appreciated the detailed explanations provided by midwives during routine examinations, although 77.3% (n=17) noted restrictions in physically interacting with the medical equipment used during these examinations (Table I).

While a substantial portion of the participants did not experience any pregnancy-related complications, 13.6% (n=3) of the women reported conditions, such as gestational diabetes mellitus and hypertension.

### Childbirth experience

The majority of the women (72.7%, n=16) gave birth after 37 completed weeks, with a high rate of cesarean section of 59.1% (n=13). In the present study, it was found that 27.3% of the participants (6 out of 22) reported giving birth to infants with a low birth weight (<2,500 g). The majority felt respected and involved in childbirth decisions, and a large proportion (68.2%, n=15) did not experience loneliness or anxiety at the time of birth (Table I).

### Puerperium period

During the puerperium period, only 1 participant reported continuing to smoke, albeit minimally at one cigarette following each breastfeeding session. The remainder of the cohort abstained from smoking, alcohol consumption and drug use during this period.

A large portion of participants, 59.1% (n=13), expressed concerns that midwives did not adequately familiarize them with the layout of the maternity room. This lack of orientation contributed to difficulties in navigating the space effectively. Furthermore, 68.2% (n=15) encountered challenges in recognizing and responding to the needs of their infants, a situation exacerbated by 81.8% (n=18) of midwives not describing the facial expressions of their infants, which would have facilitated the enhanced maternal understanding and bonding (Table I).

House visits by midwives post-birth were reported by 22.7% (n=5) of the women. In addition, 45.5% (n=10) of the participants expressed the desire for more frequent visits to support activities such as breastfeeding, including these with no visits. The participants expressed a need for midwives during the puerperium period, with comments, such as ‘as many times as necessary’, ‘until I establish breastfeeding’, and specific requests for guidance on basic caregiving tasks, such as diaper changes. Additionally, 72.7% (n=16) of the women breastfed their infants from 1 month up to 24 months, indicating a commitment to prolonged breastfeeding. From the 3 patients who suffered from glaucoma, none used teratogenic eyedrops and all breastfed their infants. Table I summarizes all the clinical and demographic characteristics of the study population.

### Attitude towards healthcare professionals and midwifery and gynecological care units

Opinions on the level of care provided by health professionals varied among the participants: Of the participants, 45.5% (n=10) felt they received the same level of care as women without visual impairments, while 54.5% (n=12) viewed the care as humanitarian. However, perceptions about the awareness of healthcare providers towards the needs of visually impaired women were mixed, with 50% (n=11) believing that midwives and gynecologists were sufficiently willing to provide appropriate care.

Notwithstanding these perceptions, 90.9% (n=20) of participants identified a substantial gap in the knowledge and specialization of medical staff regarding perinatal care for visually impaired women, advocating for enhanced training and professional development. A unanimous majority of the participants felt that comprehensive training and lifelong learning for healthcare providers are essential. They suggested that HCPs should attend specialized seminars to better understand and cater to the needs of visually impaired patients.

Participant satisfaction with the attitude of HCPs varied, with 40.9% (n=9) expressing moderate levels of satisfaction and 31.8% (n=7) expressing high levels of satisfaction. These participants appreciated when healthcare providers made efforts to overcome their lack of knowledge about dealing with disabilities. Conversely, a few women were dissatisfied with the care received, highlighting instances of poor communication and neglect that potentially endangered their health. Notably, no participant rated the midwifery and gynecological care they received as ‘excellent’, underscoring a critical area for improvement in the healthcare system. The attitude towards healthcare professionals and midwifery and gynecological care units is summarized in Table II.

## Discussion

To the best of our knowledge, the present study is first to explore the experiences of pregnancy, childbirth and the puerperium among women with visual impairments in Greece. The results presented herein underscore the critical need for equitable access to midwifery and gynecological services for all women.

Demographically, the majority of the study participants were over >30 years of age, with 86.4% (n=19) being >35 years and 9.1% (n=2) between 30 and 34 years of age. These figures align with maternal age trends previously reported in various countries, including Israel (14), USA (8,15), Iran (16,19) and Poland (6). In the present study, the majority of the participants were married (68.2%), held university degrees (68.2%), and had annual incomes either up to or above €20,000. By contrast, previous studies have indicated that women with chronic disabilities, such as blindness are more likely to be divorced or have only a high school education and are less likely to be employed or have high incomes (11,12,15,16).

A notable aspect of the present study is the etiology of visual impairment, with reported causes including glaucoma, cataract, Stargardt disease, Leber's congenital amaurosis, retinal detachment, retinoblastoma and aniridia. These are in line with the findings of previous research demonstrating that cataract, glaucoma, trachoma and onchocerciasis are the main reasons for blindness in individuals (20). This information was derived from specific medical tests documented in the medical records of the participants.

In the present study, during pregnancy, 81.8% (n=18) of the participants reported supportive attitudes from HCPs. This is in contrast to other studies where women with disabilities faced discouragement from pregnancy due to perceptions of asexuality (6,8,10,15). Among the participants in the present study, the majority of pregnancies were planned (72.7%), with a minority utilizing fertility treatments, such as IVF. The present study also noted a lower incidence of smoking and no alcohol or drug use during pregnancy among participants, a finding consistent with prior research (5). Routine prenatal examinations commenced in the first trimester for all participants, diverging from previous findings (13). Additionally, 31.8% (n=7) of the participants attended childbirth preparation classes, but some expressed dissatisfaction with their adaptation to specific needs, a sentiment that other studies have echoed (7).

As for childbirth specifics, 27.3% (n=6) of the participants in the present study reported preterm deliveries, and 59.1% (n=13) underwent cesarean sections, with the majority of newborns weighing >2,500 kg, a finding which was consistent with previous studies (5,10,13,14). In the present study, the finding of a high percentage of low-birth-weight infants aligns with several other studies focusing on pregnant women with visual impairments and disabilities. For instance, the study by Ofir *et al* (14) reported that women with visual impairments had a higher incidence of low-birth weight infants compared to the general population. Similarly, the study by Mitra *et al* (13) indicated that women with disabilities, including visual impairments, are at an increased risk of adverse birth outcomes, including low birth weight. During the puerperium, challenges included recognizing infant needs, with some women relying on non-visual cues due to inadequate support from midwives (19). This period also highlighted unmet needs for specific accommodations in hospital settings, such as information on room layouts and proximity to essential facilities.

Herein, the finding that 68.2% of the blind pregnant women did not report feelings of loneliness or anxiety at the time of birth is noteworthy and is in contrast to common perceptions about the emotional vulnerability of visually impaired individuals during major life events, such as childbirth (21). Previous research has emphasized the importance of robust support systems in mitigating feelings of loneliness and anxiety during childbirth. The presence of supportive HCPs and family members significantly influences the emotional well-being of birthing women, reducing feelings of loneliness and anxiety (22). This suggests that if visually impaired women receive effective support from healthcare providers and have family or other support networks present, their experience of childbirth could be less anxiety-provoking. In addition, according to another study, women who are better prepared for childbirth through prenatal education and childbirth preparation classes report lower levels of anxiety and a more positive childbirth experience (23). If the women in the present study participated in tailored childbirth education that addressed their specific needs as visually impaired individuals, this could have contributed to their emotional resilience during birth.

In the present study, half (50%) of the participants perceived that HCPs provided comparable levels of care to visually impaired and sighted women, though there remains room for improvement in provider knowledge and sensitivity. This issue calls for targeted interventions, such as enhanced training for HCPs, more accessible hospital design, and improved patient-provider communications, in order to improve perinatal care for women with visual impairments. There is a critical need for specialized training and adapted healthcare practices (24).

The clinical implications of the present study on the experiences of visually impaired women during pregnancy, childbirth, and the puerperium in Greece are substantial, providing several actionable insights for HCPs and policymakers There is a clear need for the additional training for doctors, nurses, midwives and other healthcare providers on the specific needs of visually impaired patients. Training should focus not only on the physical aspects of care, but also on improving communication methods, understanding accessibility issues and using appropriate assistive technologies. The study emphasizes the need to develop and provide specialized resources specifically tailored to the needs of visually impaired women. This could include informational materials in accessible formats (e.g., Braille, audio), specialized equipment for prenatal care, and modifications to existing healthcare facilities to enhance accessibility. The findings suggest a need for policy interventions that ensure equitable access to healthcare services for visually impaired women. Policies could focus on improving transportation to healthcare facilities, enhancing the physical layout of healthcare settings, and ensuring that all health communication is accessible. Clinicians should consider developing individualized care plans that consider the unique challenges faced by visually impaired women. These plans could include more frequent prenatal visits, tailored childbirth preparation classes, and specialized postpartum support. There is an imperative to integrate inclusivity into everyday clinical practice. This includes training staff to be aware of and sensitive to the stigmas and biases that visually impaired women may face, as well as directly addressing these issues in clinical settings. The unique stresses and potential isolation faced by visually impaired mothers suggest a need for enhanced mental health support during and after pregnancy. HCPs should be vigilant in monitoring the mental health of their patients and providing appropriate referrals and support services. Encouraging the development and involvement of support networks, including peer-led groups for visually impaired parents, can provide valuable emotional and practical support, enhancing the overall pregnancy and postpartum experience.

Despite its small scale, the diverse sample study sample herein suggests that the experiences of visually impaired women giving birth post-2005 in Greece share both similarities and distinct differences from global data, providing insight that may be informative for future healthcare policies and practices.

The study has some potential limitations however, which should be mentioned. These are as follows: i) Small sample size: The present study only included 22 participants, which limits the generalizability of the findings. A larger sample size would provide more robust data and allow for more definitive conclusions. ii) Geographic limitation: The present study was mainly confined to Athens, Greece. The inclusion of participants from other regions would offer a more comprehensive understanding of the perinatal care experiences of visually impaired women across the country. iii) Retrospective nature: The retrospective design relies on the recall of past events by participants, which may introduce recall bias. Prospective studies could provide more accurate and timely data. iv) Lack of a control group: The absence of a comparison group of non-visually impaired women makes it difficult to isolate the specific challenges faced by visually impaired women from broader trends in maternal healthcare. v) Self-reported data: The reliance on self-reported data can introduce bias, as participants may underreport or overreport certain behaviors or experiences due to social desirability or personal interpretation of the questions. vi) Technological accessibility: While the electronic questionnaire was designed to be accessible, it may still exclude individuals who are less comfortable with technology or lack access to the necessary devices and software. vii) Cultural and systemic factors: The findings of the present study were influenced by the specific cultural and healthcare system context of Greece, which may limit their applicability to other countries with different healthcare systems and cultural norms regarding disability. Another limitation is that the researchers do not have available representative examination tests proving the visual impairment of the participants.

In conclusion, the present study provides valuable insight into the reproductive healthcare experiences of visually impaired women in Greece, highlighting substantial barriers and societal challenges. The findings emphasize the need for tailored healthcare services that address the unique needs of this population and ensure equitable access to care. Despite a proactive approach by these women to planning pregnancies and seeking prenatal care, stigmatization and inadequate healthcare service adaptations remain pervasive. This underscores the urgent need for healthcare providers to undergo comprehensive training to better understand and address the needs of visually impaired women. In essence, the present study calls for systemic reforms in maternal healthcare practices to remove both physical and attitudinal barriers, ensuring a respectful, competent, and inclusive healthcare environment for all women, regardless of visual impairment.

## Supplementary Material

QUESTIONNAIRE

## Figures and Tables

**Table I:** Clinical and demographic characteristics of the study population.

Characteristic	No. of participants	Percentage
Age range (25-29 years)	1	4.5
Age range (30-34 years)	2	9.1
Age range (>35 years)	19	86.4
Marital status (married)	15	68.2
Marital status (single)	3	13.6
Marital status (divorced)	2	9.1
Marital status (widowed)	1	4.5
Marital status (estranged)	1	4.5
Number of children (one)	11	50.0
Educational level (higher education)	12	54.5
Employment status (employed)	9	40.9
Annual family income (€7,000-€20,000)	15	68.2
Annual family income (above €20,000)	7	31.8
Residence (urban-Athens)	16	72.7
Residence (rural)	6	27.3
Perinatal care type (public sector)	15	68.2
Perinatal care type (private sector)	7	31.8
Type of visual impairment (total blindness)	14	63.6
Type of visual impairment (partial blindness)	8	36.4
Onset of blindness (congenital)	10	45.5
Onset of blindness (later in life)	12	54.5
Retinal detachment	4	19
Stargardt disease	4	19
Incorrect handling of incubator-prematurity	4	19
Glaucoma	3	14
Congenital cataract	2	9.5
Leber syndrome	2	9.5
Aniridia	1	5
Other	1	5
Husband's disability (yes)	13	59.1
Husband's disability (no)	9	40.9
Planned pregnancy (yes)	16	72.7
Planned pregnancy (no)	6	27.3
Assisted reproduction (IVF)	2	9.1
Pregnancy outcome (miscarriage/abortion)	1	4.5
Pregnancy outcome (childbirth)	21	95.5
Smoking status (prior to pregnancy-smoker)	6	27.3
Smoking status (prior to pregnancy-non-smoker)	16	72.7
Smoking status (during pregnancy-reduced smoking)	3	13.6
Smoking Status (during pregnancy-non-smoker)	19	86.4
Alcohol consumption (before pregnancy-occasional use)	14	63.6
Alcohol consumption (before pregnancy-no use)	8	36.4
Alcohol consumption (during pregnancy-occasional use)	1	4.5
Alcohol consumption (during pregnancy-no use)	21	95.5
Drug use (none)	22	100.0
Weight change during pregnancy (5-12 kg gain)	20	90.9
Weight change during pregnancy (weight loss)	1	4.5
Weight change during pregnancy (significant gain)	1	4.5
Familial support (well-supported by husband)	15	68.2
Positive interactions with midwives (yes)	18	81.8
Prenatal care explanations (detailed)	16	72.7
Restrictions in equipment interaction (yes)	17	77.3
Pregnancy complications (yes)	3	13.6
Pregnancy complications (no)	19	86.4
Childbirth preparation classes (positive to neutral)	-	-
Gestational age at birth (>37 weeks)	16	72.7
Mode of delivery (cesarean section)	13	59.1
Infant birth weight (<2,500 g)	6	27.3
Infant birth weight (≥2,500 g)	16	72.7
Emotional experience (no loneliness/anxiety)	15	68.2
Emotional experience (loneliness/anxiety)	7	31.8
Smoking status (puerperium-continued smoking)	1	4.5
Smoking status (puerperium-abstained)	21	95.5
Alcohol consumption (puerperium-no use)	22	100.0
Drug use (puerperium-none)	22	100.0
Familiarization with maternity room (inadequate)	13	59.1
Recognizing infant needs (challenges)	15	68.2
Midwives describing infant expressions (no)	18	81.8
House visits by midwives (yes)	5	22.7
House visits by midwives (no)	17	77.3
Desire for more frequent visits (yes)	10	45.5
Duration of breastfeeding (1 to 24 months)	16	72.7

**Table II:** Attitude towards healthcare professionals and midwifery and gynecological care units.

Category	No. of participants	Percentage
Perception of care quality		
Same level of care as women without impairments	10	45.5
Care viewed as humanitarian	12	54.5
Awareness of healthcare providers		
Sufficient willingness to provide appropriate care	11	50
Perceived knowledge gap in healthcare professionals		
Identified substantial knowledge gap	20	90.9
Advocacy for enhanced training		
Support for comprehensive training and lifelong learning	22	100
Participant satisfaction with attitude of healthcare professionals		
High satisfaction	9	40.9
Moderate satisfaction	7	31.8
Rating of care quality		
Excellent care	0	0

## Data Availability

The datasets used and/or analyzed during the current study are available from the corresponding author on reasonable request.
