# Supplementary material for: Exploring perinatal care and birth experiences in women with visual impairment: A retrospective study
Source: Med Int (Lond). 2024 Aug 23;4(6):66. doi: 10.3892/mi.2024.190 (PMC11384187; doi:10.3892/mi.2024.190)
Supplement: QUESTIONNAIRE [file Supplementary_Data.pdf]

Data S1

QUESTIONNAIRE

SECTION 1-DEMOGRAPHICAL AND CLINICAL CHARACTERISTICS

1. At which age category do you belong to?
a. <20 years
b. 20-24 years
c. 25-29 years
d. 30-34 years
e. >35 years

2. Nationality
a. Greek
b. Other

3. Marital status
a. Single
b. Married
c. Divorced
d. Widowed
e. Estranged

4. Educational level
a. Primary school
b. High school
c. Bachelor's degree
d. Master's degree
e. Doctorate

5. Profession
a. State employee
b. Private employee
c. Freelance
d. Household
e. Unemployed
f. College student

6. Cause of impaired vision
a. Glaucoma
b. Cataract
c. Increased eye pressure
d. Aniridia
e. Other

7. At what age did you give your first birth?
8. At what age did the visual impairment develop?
a. Congenital
b. Other

9. Number of living children
10. Has your partner/husband also an impaired vision of any other type of disability?
a. Yes
b. No

SECTION 2-PREGNANCY PERIOD

11. How many times have you been pregnant?
a.1
b. 2
c. Other

12. How many times have you given birth?
a. 1
b. 2
c. Other

13. Was the pregnancy planned?
a. Yes
b. No

14. Was any method of assisted reproduction used? (IVF, insemination)
a. Yes
b. No

15. Year of last pregnancy
16. Did you smoke during pregnancy?
a. Every day or more often
b. 2-6 times a week
c. Once a week
d. Once a month
e. Never
f. Other

17. Did you consume alcohol during pregnancy?
a. Every day or more often
b. 2-6 times a week
c. Once a week
d. Once a month
e. Never
f. Other

18. Did you use drugs during pregnancy?
a. Every day or more often
b. 2-6 times a week
c. Once a week
d. Once a month
e. Never
f. Other

19. Weight you had before pregnancy
20. Weight you had at the end of pregnancy
21. The pregnancy/pregnancies were:
a. Single
b. Twin
c. Other

22. To what extent was your partner/husband supportive of your pregnancy news?
a. 1-Not at all
b. 2-Slightly
c. 3-Moderately
d. 4-Very
e. 5-Extremely

23. Did you receive sexual, physical or domestic abuse during pregnancy from members of your family?
a. 1-Not at all
b. 2-Slightly

c. 3-Moderately
d. 4-Very
e. 5-Extremely

24. How would you characterize the attitude of health professionals (midwives) to the news of pregnancy?
a. Supportive
b. Critical
c. Neutral
d. Other

25. In what trimester of pregnancy did you start the routine tests?
a. 1st trimester
b. 2nd trimester
c. 3rd trimester

26. During the routine examinations, did the midwives (and other health professionals) explain to you the procedure of each examination?
a. Yes
b. No

27. Were you given the opportunity to touch with your hand (touch activation) the machine that would be used for the examination?
a. Yes
b. No

28. Did you attend classes on painless childbirth-parenthood preparation?
a. Yes
b. No

29. To what extent were the painless childbirth-parenthood preparation courses adapted to your needs/expectations? (enable touch)
a. 1-Not at all
b. 2-Slightly
c. 3-Moderately
d. 4-Very
e. 5-Extremely

30. Did you feel insecure before pregnancy?
a. Always
b. Usually
c. Sometimes
d. Rarely
e. Never

31. Complications during pregnancy
a. Diabetes before pregnancy
b. Gestational Diabetes Mellitus
c. Gestational Hypertension
d. Vaginal Hemorrhage
e. Placenta suppository
f. Hyperemesis gravidarum
g. Other

32. When you had questions about the changes you felt in your body, did the midwife explain in a way that you could understand?
a. Yes
b. No

SECTION 3-CHILDBIRTH

33. When did the delivery take place?
a. <37 weeks of gestation
b. ≥37 weeks of pregnancy

34. Kind of Childbirth
a. Natural Childbirth (Vaginal)
b. Cesarean section

35. Year of last childbirth

36. Sex of newborn
a. Male
b. Female

37. Newborn Birth Weight
a. ≥2,500 gr
b. <2,500 gr
c. Other

38. At what extent were you actively involved in the decisions at the time of your birth? (e.g. in the case of epidural anesthesia when and if you requested it)
a. 1-Not at all
b. 2-Slightly
c. 3-Moderately
d. 4-Very
e. 5-Extremely

39. In the delivery room, did the health professionals (midwife/obstetrician, gynecologist, anesthesiologist) treat you with respect?
a. Yes
b. No

40. At what extent did you feel alone or stressed during the hours you were in the delivery room?
a. 1-Not at all
b. 2-Slightly
c. 3-Moderately
d. 4-Very
e. 5-Extremely

SECTION 4-PUERPERIUM PERIOD

41. Did you smoke during the puerperium period?
a. Every day or more often
b. 2-6 times a week
c. Once a week
d. Once a month
e. Never
f. Other

42. Did you consume alcohol during the puerperium period?

a. Every day or more often
b. 2-6 times a week
c. Once a week
d. Once a month
e. Never
f. Other

43. Did you use drugs during the puerperium period?

a. Every day or more often
b. 2-6 times a week
c. Once a week
d. Once a month
e. Never
f. Other

44. Did the midwives show you how the maternity room was set up? (e.g. where the toilet was)
a. Yes
b. No

45. Did you find it difficult to realize the needs of your newborn? (if he's hungry, if he's in pain)
a. Yes
b. No

46. How many times did a midwife visit you at home during the puerperium period?
47. How many times would you like the midwife to visit you at home during puerperium period?
48. How long did the breastfeeding last?

SECTION 5-HEALTH PROFESSIONAL AND CARE EVALUATION

49. To what extent did you receive the same level of obstetric and gynecological care as women without visual impairment?
a. 1-Not at all
b. 2-Slightly
c. 3-Moderately
d. 4-Very
e. 5-Extremely

50. To what extent was the care you received from health professionals (midwives, gynecologists) humane?

a. 1-Not at all
b. 2-Slightly
c. 3-Moderately
d. 4-Very
e. 5-Extremely

51. To what extent do the midwives understand the needs of visually impaired people?
a. 1-Not at all
b. 2-Slightly
c. 3-Moderately
d. 4-Very
e. 5-Extremely

52. Midwives/obstetricians and gynecologists are not willing to provide obstetric and gynecological care to visually impaired women
a. 1-Not at all
b. 2-Slightly
c. 3-Moderately
d. 4-Very
e. 5-Extremely

53. To what extent is the participation of health professionals in educational seminars (continuing education) necessary to provide personalized and holistic care to women with visual impairment?
a. 1-Not at all
b. 2-Slightly
c. 3-Moderately
d. 4-Very
e. 5-Extremely
